# Classification and identification of tea diseases based on improved YOLOv7 model of MobileNeXt

**DOI:** 10.1038/s41598-024-62451-y

**Published:** 2024-05-23

**Authors:** Yuxin Xia, Wenxia Yuan, Shihao Zhang, Qiaomei Wang, Xiaohui Liu, Houqiao Wang, Yamin Wu, Chunhua Yang, Jiayi Xu, Lei Li, Junjie He, Zhiyong Cao, Zejun Wang, Zihua Zhao, Baijuan Wang

**Affiliations:** 1 College of Mechanical and Electrical Engineering, Yunnan Agricultural University, Kunming, 650201 China; 2 College of Tea Science, Yunnan Agricultural University, Kunming, 650201 China; 3 College of Information Engineering, Yunnan Agricultural University, Kunming, 650201 China

**Keywords:** Tea leaf disease classification identification, Improved YOLOv7, MobileNeXt, Dual-layer routing attention mechanism, Computational biology and bioinformatics, Computational models, Computational platforms and environments, Image processing, Machine learning

## Abstract

To address the issues of low accuracy and slow response speed in tea disease classification and identification, an improved YOLOv7 lightweight model was proposed in this study. The lightweight MobileNeXt was used as the backbone network to reduce computational load and enhance efficiency. Additionally, a dual-layer routing attention mechanism was introduced to enhance the model’s ability to capture crucial details and textures in disease images, thereby improving accuracy. The SIoU loss function was employed to mitigate missed and erroneous judgments, resulting in improved recognition amidst complex image backgrounds.The revised model achieved precision, recall, and average precision of 93.5%, 89.9%, and 92.1%, respectively, representing increases of 4.5%, 1.9%, and 2.6% over the original model. Furthermore, the model’s volum was reduced by 24.69M, the total param was reduced by 12.88M, while detection speed was increased by 24.41 frames per second. This enhanced model efficiently and accurately identifies tea disease types, offering the benefits of lower parameter count and faster detection, thereby establishing a robust foundation for tea disease monitoring and prevention efforts.

## Introduction

Tea culture has a rich history and culture and a huge global demand. With the improvement of public health awareness, tea was widely popular because of its inherent antioxidant properties and potential health advantages^1^. Abundant in polyphenols and antioxidants, tea contributes to the neutralization of free radicals, mitigates oxidative stress, and promoting cellular well-being^2^. China is the world’s largest tea consumer, and its tea production ranks first in the world. Yunnan has become one of the provinces with the most abundant biodiversity in China due to its diverse climate and topography, and has bred a unique plateau mountain tea industry^3^. Due to the long-term warm and humid environment, it provids the environment and conditions for the infection of tea diseases, showing the characteristics of a wide variety and difficult prevention and control, resulting in a decline in tea yield and quality. The growth of tea trees is often marred by the frequent occurrence of diseases, which exert a considerable detrimental impact on the tea industry^4^. Disease is not only the reduction of yield and quality^5^, as it also inflicts severe economic losses upon tea farmers and producers. In order to reduce losses, timely and accurate identification of tea diseases has become a crucial task to ensure tea plantation management and sustainable development.

In the realm of traditional target detection algorithms, reliance primarily rests on the analysis of color, texture, and related attributes^6^. Such methods predominantly express the shallow features while falling short^7^ in their capacity to effectively extract more deeper features. In complex scenes, there are problems such as low robustness and poor generalization, which is difficult to meet the needs of accurate identification of diseases.

In recent years, machine learning and image processing technology have developed rapidly. Using machine learning and image processing technology has been widely used in the field of crop research^8–10^. Maria et al.^11^ firstly used k-means clustering method to extract relevant features of cauliflower diseases, and used random forest and multiple NN models to compare the classification accuracy. Finally, the model accuracy of InceptionV3 was better, reaching 90.08%; Monu Bhagat et al.^12^ fused LBP and VGG-16 models to identify sweet pepper diseases, and the detection effect was good. At the same time, deep learning technology has developed rapidly and has been widely used in crop research^13–15^. Soeb et al.^16^ proposed a tea disease detection model based on YOLOv7, and the recognition results of rust spot and other diseases in a simple background reached more than 95%, and the detection effect was good. Zhang et al.^17^ proposed the ShuffleNetv2-YOLOv5-Lite-E edge device detection method to identify one bud and two leaves of tea. The lightweight backbone network and pruning algorithm are used to improve the accuracy and meet the deployable requirements; Chen J et al.^18^ proposed the Mobile-DANet network architecture, using the depth-wise separable convolution to replace the traditional convolution layer, and then embedding the attention module to learn the relationship between channels. Under complex background conditions, the average accuracy of identifying corn crop diseases also reached 95.86%. Lee et al.^19^ used the convolutional neural network Faster R-CNN model to identify and detect tea diseases, and the detection accuracy rate reached 77.5%.

In summary, there were some problems in the existing research, such as the data background being relatively single, the model calculation being more complex, the model calculation load being large, and the deployment being difficult. Therefore, this paper took the datasets of bituminous coal disease, zonate spot disease, anthrax, and leaf blight in complex scenes as the research object and improved the model as follows on the basis of the YOLOv7 model. Firstly, the MobileNeXt inverted residual structure^20,21^ was used to replace the backbone network of the original YOLOv7 to reduce the overall parameters of the model and achieve the purpose of lightweighting the model, which laid a foundation for the further deployment and application of the recognition model. Secondly, in order to achieve more flexible computing resource allocation and feature perception, a dual-layer routing attention mechanism^22,23^ module was introduced to enhance the ability to capture the detailed information features of tea diseases in complex environments, which could effectively improve the robustness and generalization ability of the model. Finally, the loss function was replaced by SIoU^24^ for optimization. The advantage was that the angle factor was added to make the prediction box closer to the real box faster, reduce the regression loss, and accelerate the convergence speed of the model. The purpose of this study was to propose a model for disease classification and recognition under the complex background of tea garden, which had low computational complexity and high recognition accuracy^25^, so as to provide a reliable basis for disease prevention and control of tea garden.

## Methods

### Tea disease image dataset

The image dataset employed in this study for tea disease analysis was procured from tea gardens located at Yunnan Agricultural University in Kunming, Yunnan Province, and the Hekai tea garden base in Menghai County, Xishuangbanna Prefecture. These images were captured during two distinct periods, namely mid-November 2022 and mid-June 2023. To ensure the richness and diversity of the dataset for robust model performance in target detection, data acquisition was carried out under varying light intensities and weather conditions. Notably, the images were acquired within natural environmental conditions, without imposing restrictions on imaging distances, using an OPPO R15 mobile phone as the acquisition device. In total, 1823 image datasets were amassed, featuring a resolution of 4608 pixels by 3456 pixels, and were originally stored in JPG format.

**Figure 1:**
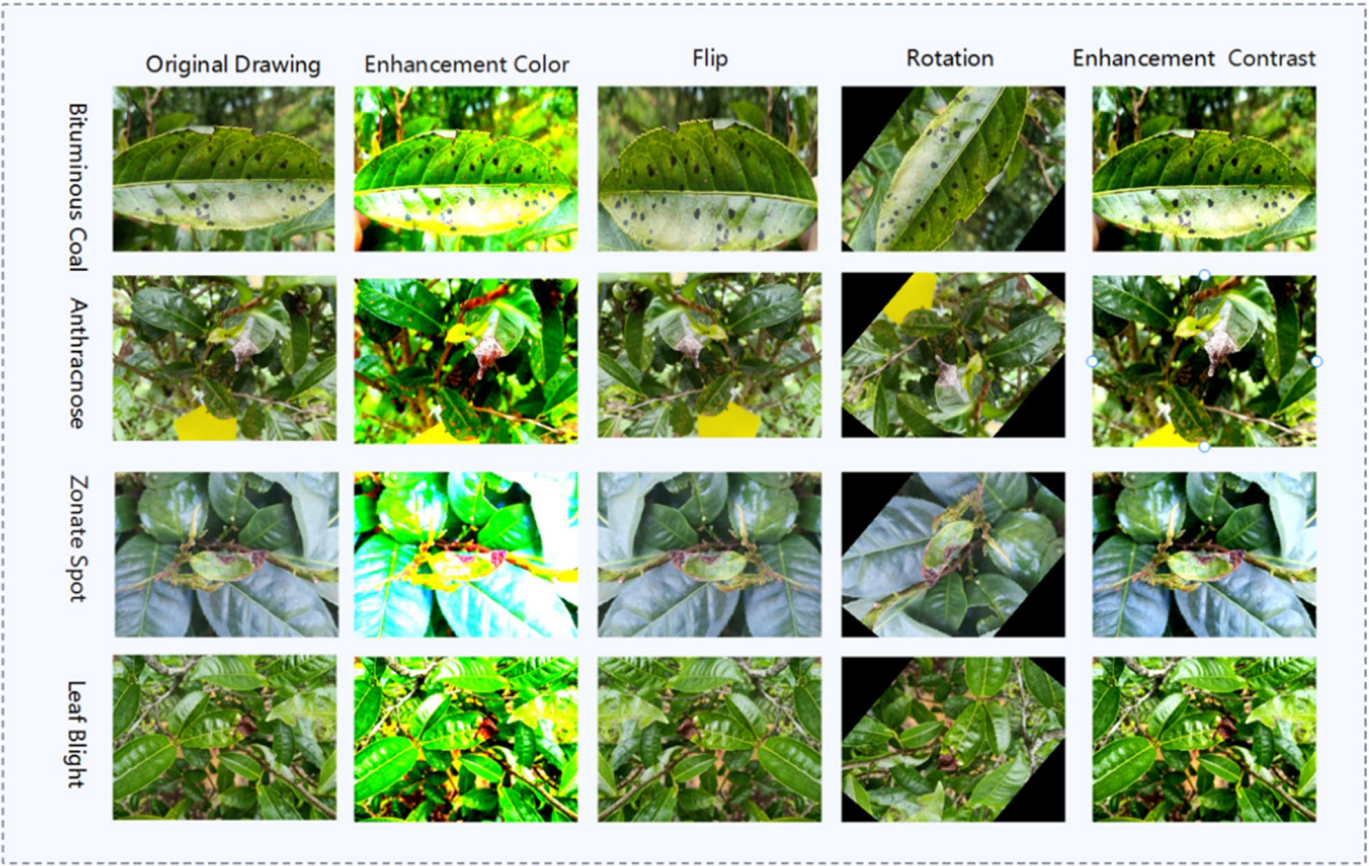
Data enhancement contrast diagram.

During the collection of tea disease data, the four diseases, namely bituminous coal disease, anthracnose, zonate spot, and leaf blight, were distinctly classified, and redundant data were systematically removed. This refinement process resulted in the selection of 231, 293, 145, and 187 high-quality initial disease datasets for each respective condition. To mitigate the risk of over-fitting during the model training process, attributed to the limited sample size within the image dataset, this study judiciously employed data augmentation techniques, including enhancement color, flipping, random rotation and enhancement contrast as shown in Fig. 1. These strategies were systematically implemented to expand the dataset in a balanced and reasoned manner. Following these enhancements, the total number of images for the four types of diseases reached a substantial count of 4280.

In this investigation, the labeling of tea disease images was meticulously executed using the LabelImg manual annotation tool, and the resultant labels were subsequently organized into XML and TXT file datasets. Notably, throughout the labeling process, emphasis was placed on positioning the tea disease segments within the center of the bounding boxes. The aforementioned datasets were subsequently partitioned into training, testing, and validation sets following a ratio of 6:2:2. This partitioning resulted in a training set comprising 2568 samples, a test set consisting of 856 samples, and an equally sized validation set of 856 samples. These datasets were instrumental in the training and evaluation of the model in this study.

### Improved YOLOv7 algorithm

This paper chose to build a model based on YOLO series algorithms. YOLO series algorithms were widely used in the field of industrial development due to their advantages of simplified structure^26^, high performance, and easy deployment. Among them, YOLOv7 is one of the advanced algorithms of YOLO series, and its detection accuracy and speed were higher than other YOLO series models^27^. YOLOv7 adopts a single-stage target detection method. Its overall network structure^28^ included four parts: Input (input), Backbone (backbone network), Neck (neck), and Head. The input image is first normalized to meet the model input requirements, and then the backbone network extracts features. Finally, the extracted feature map is processed to output the predicted bounding box position and category information to better capture targets of different sizes. This method processed and obtained the target area, location, and category of the corresponding object through regression. Compared with the traditional two-stage target detection algorithm, it significantly improvs the detection efficiency while maintaining the accuracy of the target detection, which lays a foundation for the accurate detection of tea leaf diseases in complex environments.

### MobileNeXt lightweight network

In the original backbone network CSPDarknet53 of YOLOv7, a large number of ordinary convolutions are used for feature extraction, resulting in a large volume of network model parameters, which increases the computational complexity and affects the model's detection speed. Moreover, lightweight mobile devices that require memory size are also difficult to deploy. Therefore, this paper adopts a more lightweight MobileNeXt network structure instead of the original YOLOv7 backbone network. As a lightweight neural network structure, MobileNeXt can not only efficiently extract features from images in complex background environments and improve the accuracy of target detection, but also its lightweight design means that it has faster inference speed and can be well adapted to mobile devices with limited resources, ensuring its high performance without sacrificing efficiency. The overall network structure of MobileNeXt is stacked by multiple Sandglass Block modules. The module is designed according to the inverse residual structure and uses a deep separable convolution method (Dwise) to achieve greater efficiency and lightweight. The MobileNeXt backbone network structure is shown in Fig. 1. When replacing the backbone network, it is necessary to ensure that the feature map size and the number of channels of the input neck network are unchanged.

The Sandglass^29^ Block represents an hourglass residual module, characterized by its distinctive placement of depth-wise convolutions at both the outset and conclusion. This architectural choice is deliberate and seeks to maintain the spatial depth convolution layer of the channel. By interleaving pointwise convolutions between the two depth convolution layers, the aim is to augment the channel count, thus ensuring that the depth convolution transpires within a high-dimensional space, thereby enriching the expression of features. Simultaneously, the manipulation of larger channel tensors by means of depth kernels serves to significantly curtail the parameter count, achieving the objective of lightweight design. The specific formula is as follows:

1 $$\hat{G} = \emptyset_{1,p} \emptyset_{1,d} FG = \emptyset_{1,d} \emptyset_{2,p} \hat{G} + F$$

where ${\varnothing }_{i,p}$, denotes a 1 $\times$ 1 convolution and ${\varnothing }_{i,d}$ denotes a deep convolution, F denotes the input tensor ,G denotes the output tensor.

**Figure 2:**
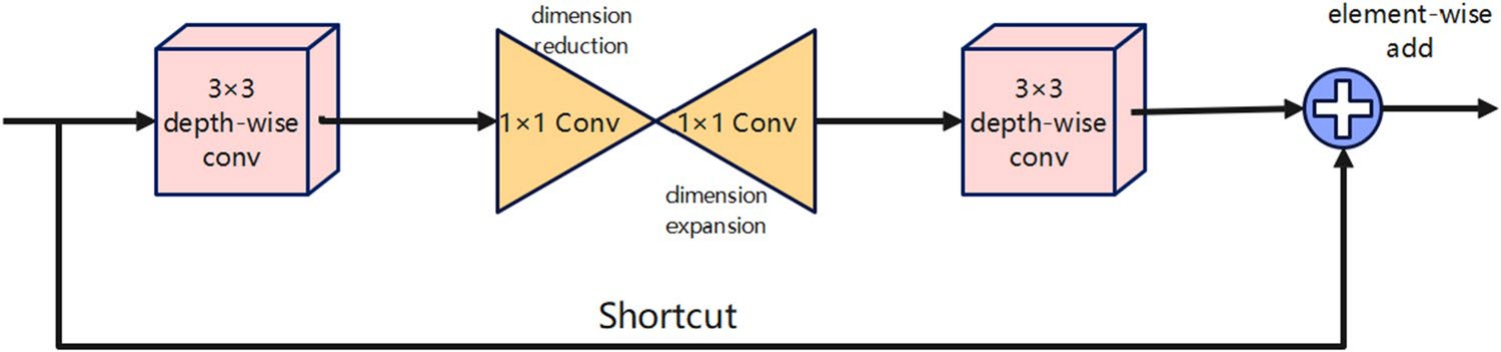
Sandglass network structure.

The Sandglass Block structure as shown in Fig. 2 adeptly capitalizes on the strengths of high-dimensional network convergence acceleration while harnessing the computational benefits offered by deep separable convolution. Through the incorporation of deep convolution layers functioning within high-dimensional space, it facilitates the acquisition of more intricate feature expressions. This approach also effectively diminishes the memory access demands associated with high-dimensional feature maps, thereby enhancing execution efficiency.

Furthermore, the Sandglass Block design guarantees the efficient transmission of a greater volume of information from the lower layers to the upper layers, bolstering gradient backpropagation. By introducing two depth-wise separable convolutions, it further encodes additional spatial information, thereby contributing to the overall efficacy of the structure.

In convolutional neural networks, the pooling layer is a commonly employed technique to reduce the dimensionality of image features, thereby enhancing classification accuracy, processing speed, as well as the model’s training speed and generalization capacity. The average pooling layer functions by segmenting the input feature map into uniform regions, subsequently down-sampling the feature map, and computing the average value of the constituent elements within each region. The resulting average value is then assigned as the output value of that respective region, thereby diminishing the size of the feature map and the number of parameters. This process effectively trims the feature map size while retaining the essential feature information. The specific computational procedure is delineated as follows:

Firstly, the input data and the size of the pooling kernel are defined^30^. It is assumed that the input data are $X\in {R}^{H*W}$, the size of the pooling kernel is k $\times$ k, and the step size is s. Then we define the output characteristic graph. The size of the output characteristic graph $Y\in {R}^{{H}^{-1}*{W}^{-1}}$:

2 $$H^{\prime} = \left[ {\frac{H - k}{s}} \right] + 1, W^{\prime} = \left[ {\frac{W - k}{s}} \right] + 1$$

The average pooling pool (X) is defined as:

3 $$pool\left( x \right)_{i,j} = \frac{1}{{k^{2} }}\mathop \sum \limits_{m = 0}^{k - 1} \mathop \sum \limits_{n = 0}^{k - 1} X_{i*s + m,j*s + n}$$

### Visual converter of the dual-layer routing attention mechanism

To bolster the model’s proficiency in extracting and representing target features, particularly when confronted with intricate scenarios and diminutive targets, this study introduced a visual converter of dual-layer routing attention mechanism. This mechanism combines the self-attention mechanism from Transformers^31^ with the feature pyramid network of BiFPN^32^ (Bi-directional Feature Pyramid Network) to elevate target detection performance. Notably, this mechanism excels in bidirectional encoding, allowing it to incorporate both global and local information. Its fundamental concept involves the selection of pertinent key-value pairs and the judicious retention of a limited portion of the routing area. This retention facilitates the preservation of fine-grained information while concurrently reducing computational demands. The Bi-level Routing Attention (BRA) mechanism encompasses several components. Initially, the feature map is subdivided into non-overlapping regions of size S $\times$ S and subjected to linear mapping, as described by the following formula:

4 $$Q = X^{r} W^{q} ,K = X^{r} W^{k} ,V = X^{r} W^{v}$$

Subsequently, the attention weight is computed for the coarse-grained Tokens, and solely the top-k regions are selected as relevant regions for engagement in fine-grained operations. This procedure is mathematically articulated as follows:

5 $$A^{r} = Q^{r} \left( {K^{r} } \right) ^{T}$$

6 $$I^{r} = topkIndex\left( {A^{r} } \right)$$

Lastly, the most pertinent top-k coarse-grained region for each Token is employed as both the key and value for involvement in the ultimate operation. To accentuate locality, a deep convolution is applied to the value. The associated formula is expressed as follows:

7 $$K^{g} = gather\left( {K,I^{r} } \right)$$

8 $$V^{g} = gather\left( {V,I^{r} } \right)$$

9 $$O = Attention\left( {Q,K^{g} ,V^{g} } \right) + LCE\left( V \right)$$

**Figure 3:**
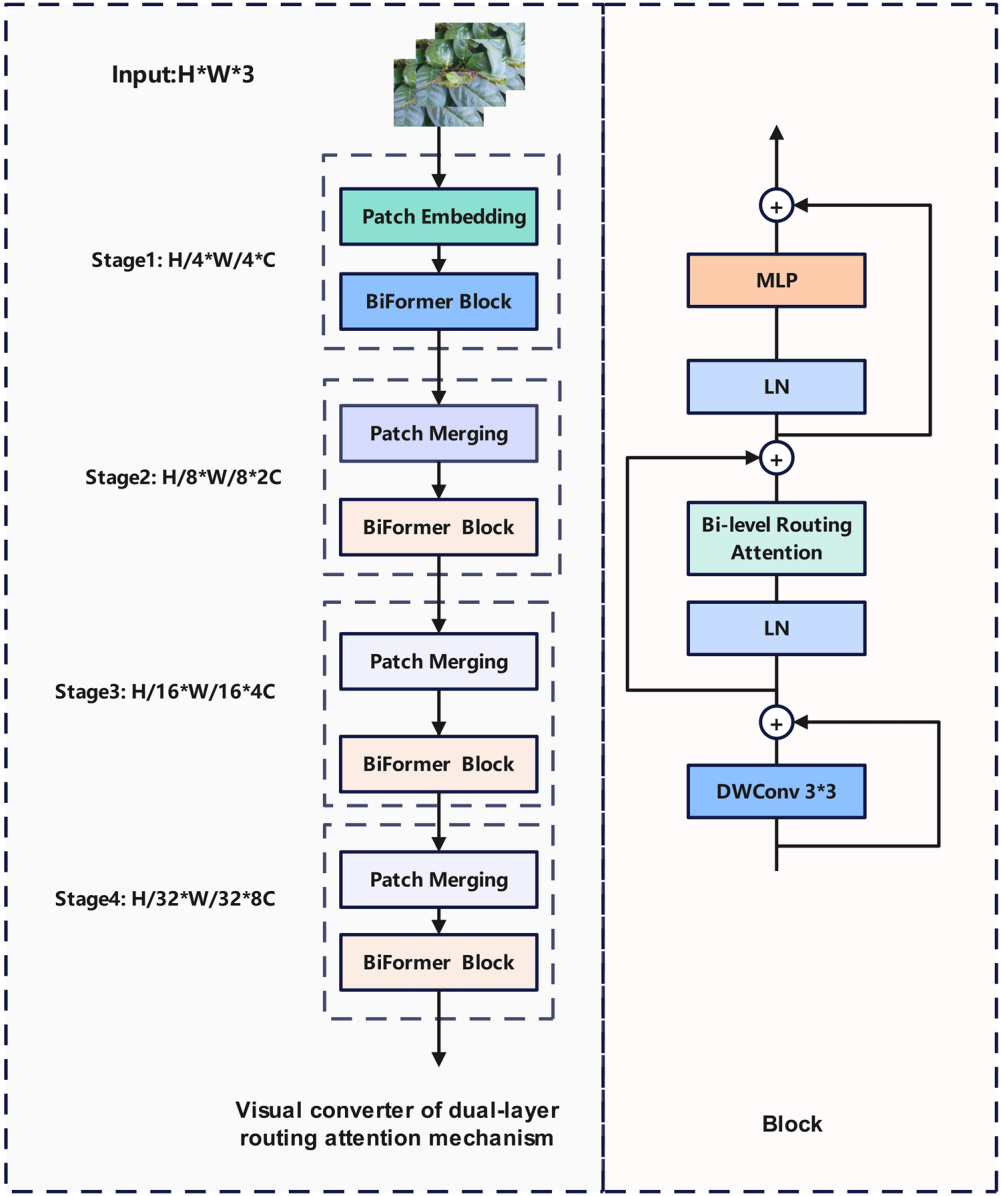
Structure diagram of attention mechanism of dual-layer routing.

Building upon the aforementioned BRA mechanism, the initial phase adopts overlapping Patch embedding. Subsequently, the second to fourth stages augment the channel count and implement patch merging blocks to downsize the input spatial resolution. This sequence is followed by feature transformation, effectively diminishing the computational load of the model and enhancing its operational efficiency. The specific structure is shown in Fig 3.

### Loss function

IoU (Intersection over Union) represented the intersection over union between the real box and the prediction box^33^, which was an important tool for measuring the distance between the prediction information and the target information. In the YOLOv7 algorithm, the CIoU loss function is used to calculate the positioning loss. The precise mathematical formulation is presented as follows:

10 $$L_{CI0U} = 1 - IoU + \frac{{\rho^{2} \left( {b,b^{gt} } \right)}}{{c^{2} }} + \alpha v$$

11 $$\alpha = \frac{v}{1 - IoU + v}$$

12 $$v = \frac{4}{{\pi^{2} }}\left( {\arctan \frac{{w^{gt} }}{{h^{gt} }} - \arctan \frac{w}{h}} \right)^{2}$$

In these equations, the variables are as follows: “b” represents the predicted box, ${b}^{gt}$ the true box, ${\rho }^{2}\left(b,{b}^{gt}\right)$ signifies the Euclidean distance between the center points of the predicted box and the true box, “c” denotes the diagonal distance of the minimum enclosing region that can encompass both the predicted box and the true box, “v” represents the aspect ratio consistency, and “α” serves as the balance parameter. However, when CIoU set the width-height ratio of the border to be equal, it led to the partial failure of the width-height ratio.

In addition, the width-height change trend of CIoU usually showed a negative correlation, that is, when the bounding box of one dimension increased, the other dimension decreased, and the problem of target prediction box mismatch was prone to occur when the bounding box was predicted. The above two points led to the mismatch between the prediction box and the real box of the model, which made the convergence effect of CIoU decrease, and the effect of disease detection in complex background was also greatly reduced.

In order to solve these two problems, this paper introduced the SIoU loss function^34^ to optimize the model. Compared with other loss functions, SIoU considered the angle factor, so that the prediction box could approach the real box faster and accelerate the convergence speed of the model. In addition, when calculating the width-height relationship between the real frame and the prediction frame, SIoU adopted the method of separate calculation, rather than the method of calculating its relative proportion, which avoided the situation that the width-height ratio of the prediction frame and the real frame was equal. The SIoU loss function included three parts: angle loss, distance loss, and shape loss. The specific calculation method is as follows:

13 $$\Lambda = 1 - 2sin^{2} \left( {arcsin\left( x \right) - \frac{\pi }{4}} \right)$$

14 $$\Delta = \mathop \sum \limits_{t = x,y} \left( {1 - e^{{ - \gamma p_{t} }} } \right)$$

15 $$\Omega = \mathop \sum \limits_{t = w,h} \left( {1 - e^{{ - w^{t} }} } \right)^{\theta }$$

In the above formula, ʌ is the angle loss; Δ is the distance loss considering the angle loss; Ω is the shape loss. In summary, the SIoU loss function is defined as follows:

16 $$L_{SIoU} = 1 - IoU + \frac{\Delta + \Omega }{2}$$

### The specific structure of the improved YOLOv7

**Figure 4:**
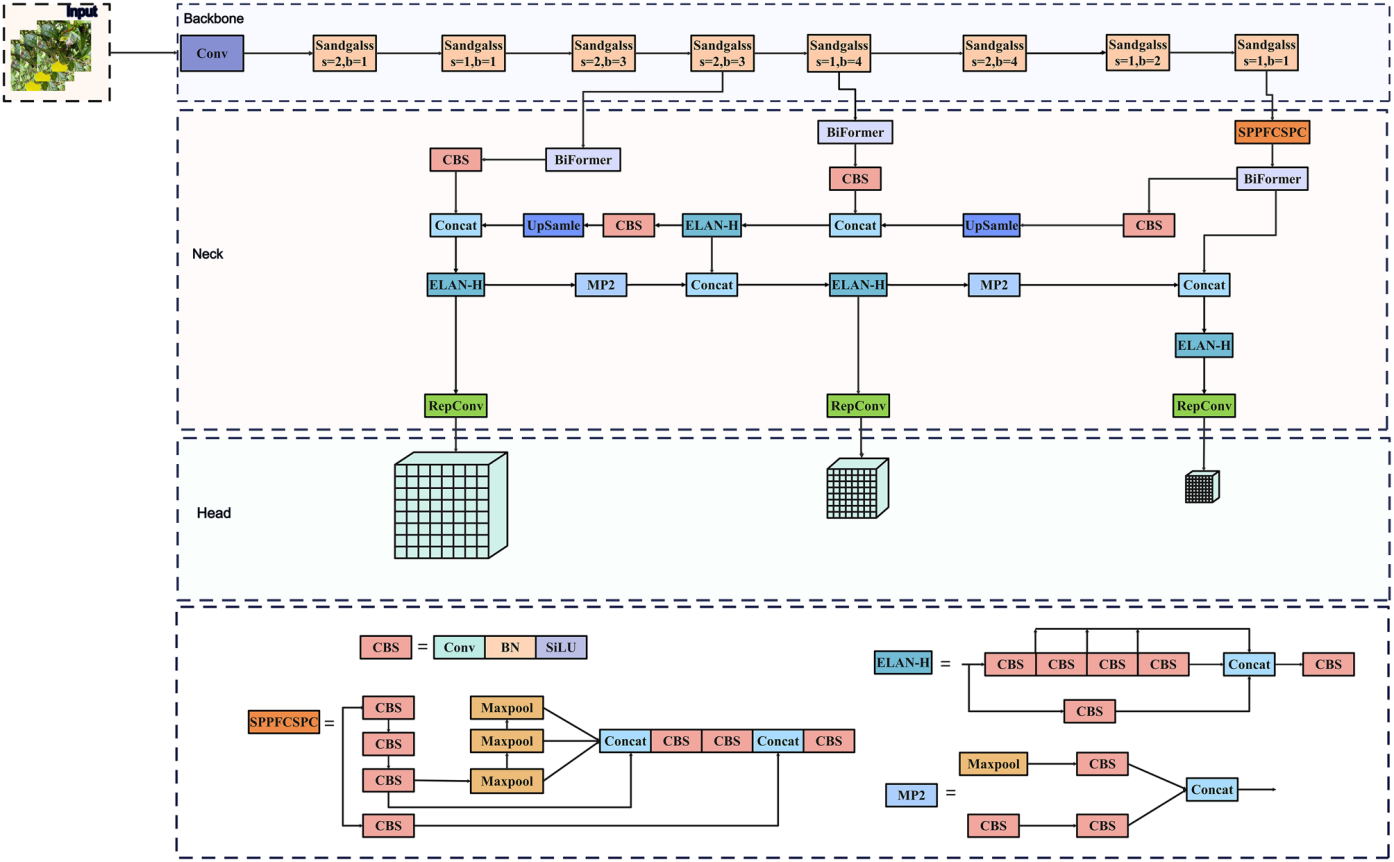
Structure diagram of improved YOLOv7.

By implementing the aforementioned refinements to the YOLOv7 model, we achieved model lightweighting and an improved capacity for extracting disease features, resulting in enhanced convergence speed. The specific network structure is depicted as shown in Fig. 4:

### Selection of evaluation indicators

In assessing the performance of target detection category models, the P value, R value, and mAP are typically chosen evaluation metrics. Here, the area under the Recall-Precision curve (AP) is pivotal^35^, and mAP serves as the average of individual category AP values, offering a comprehensive evaluation metric. The mathematical representation are articulated as follows:

17 $${\text{P}} = \frac{{\text{TP }}}{{{\text{TP}} + {\text{FP}}}}$$

18 $${\text{R}} = \frac{{{\text{TP}}}}{{{\text{TP}} + {\text{FN}}}}$$

19 $${\text{mAP}} = \mathop \smallint \limits_{0}^{1} {\text{P*}}\left( {\text{R}} \right){\text{dR}}$$

In this context, TP (True Positives) represents the count of accurately identified samples, FP (False Positives) corresponds to the number of incorrectly identified positives, and FN (False Negatives) signifies the count of missed samples.

### Experimental environment

In this study, the training model operated on the Windows 11 operating system, utilized an Intel(R) Core (TM) i9-12900H 2. 50 GHz processor, 16 GB of memory, and an NVIDIA GeForce RTX 3060 graphics card. The deep learning framework employed was PyTorch, integrated within a CUDA 11.1 virtual environment, and made use of CUDNN 8.0. The programming language utilized was Python 3.7.

## Results

In order to further compare the advantages of MobileNeXt as a backbone network, this paper replaced the backbone network of YOLOv7 with GhostNetV2, MobileNetV3, EfficientNet, and MobileNeXt. They were trained and compared them on the same data set. The performance of the model was shown in Table 1. The experimental results showed that after replacing the CSPDarknet53 module with MobileNeXt, the Precision of the improved YOLOv7 model increased by 5.06% and the Recall increased by 2.16%. The mAP increased by 2.91%, the overall parameter quantity of the model was also reduced by 12.88 M, and the frame rate was increased by 24.41 frames per second. Compared with the other three network structures of the improved YOLOv7 model, the number of total parameters was reduced by 11.06 M, 11.74 M, and 8.29 M respectively, and the Precision was increased by 9.4%, 4.82%, and 5.3% respectively. The frame rate and GFLOPs were also significantly improved, which proved that the target detection performance of the improved model using MobileNeXt as the backbone network was significantly improved, achieving lightweight and rapid detection.

**Table 1:** Comparison of results of different models.

Model	Precision	Recall	mAP	Weights	FPS	GFLOPs (G)	Total param/M
YOLOv7	0.89	0.88	0.895	74.8	41.96	105.2	37.21
YOLOv7-GhostNetV2	0.855	0.861	0.877	52.99	44.74	41.9	26.15
YOLOv7-MobileNetV3	0.892	0.869	0.893	50.7	56.0	41.4	25.47
YOLOv7-EfficientNet	0.888	0.884	0.906	58.88	52.59	20.8	28.98
YOLOv7-MobileNeXt	0.935	0.899	0.921	50.11	66.37	37.7	24.33

**Figure 5:**
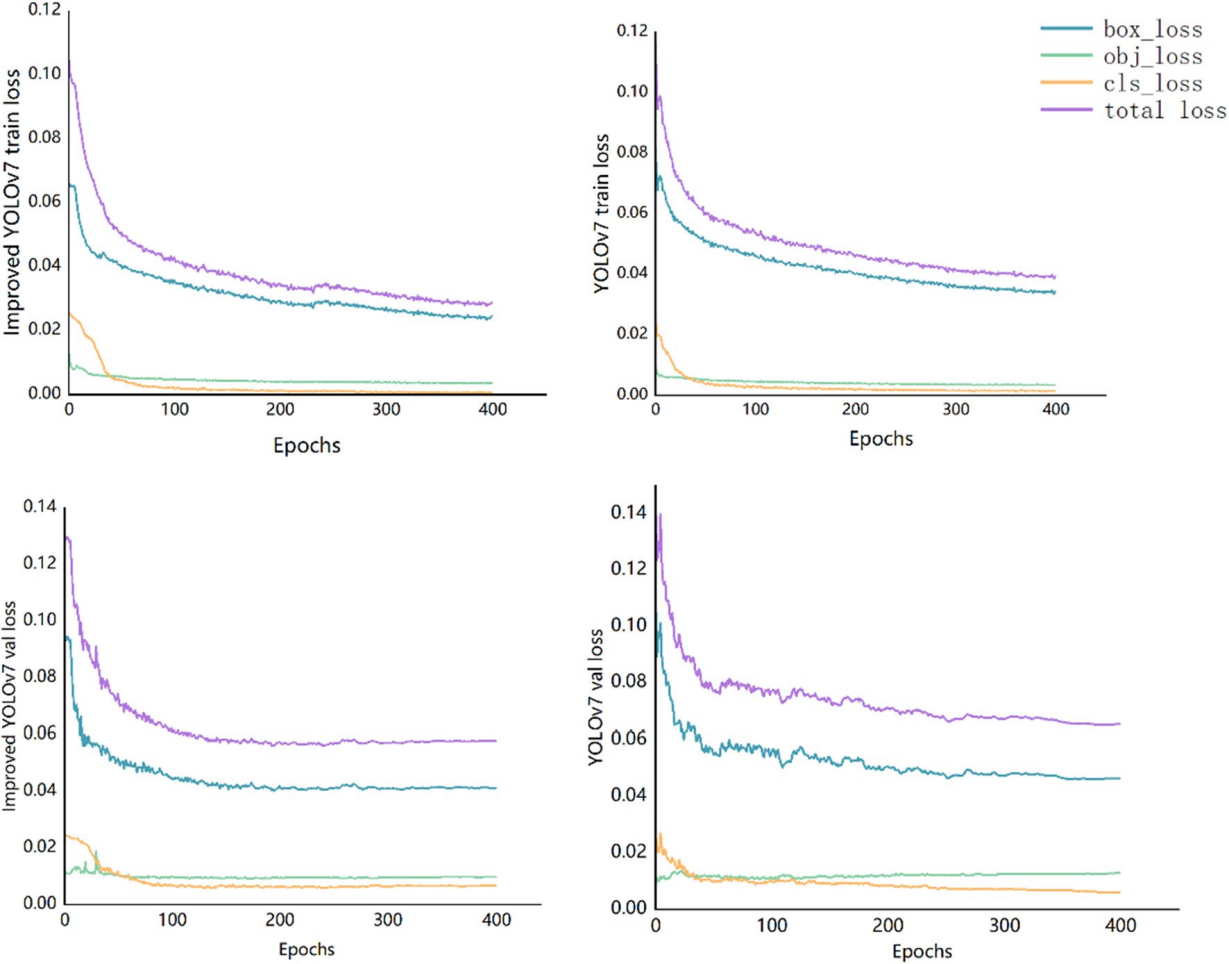
Comparison of loss function curves.

The loss function serves a dual purpose as a vital metric for quantifying the disparity between predicted outcomes and actual results, and as a fundamental criterion for appraising model quality. A smaller loss function value signifies a closer alignment between the model’s predictions and the actual results, indicative of superior model performance. In this study, the improved YOLOv7 model underwent training, with a total of 400 training iterations (Epochs). As illustrated in the subsequent figure, it is evident that during the initial stages of training for the improved YOLOv7 model, the loss function exhibits a more rapid descent in gradient speed compared to the original YOLOv7 model. Simultaneously, as training progresses, after approximately 150 epochs, there is a notable deceleration in the decline rate of the loss function. As shown in Fig. 5, by the 300 epochs, it becomes apparent that the loss curve gradually stabilizes, and the loss function commences convergence. The final results revealed that the improved model consistently maintains an overall loss value of approximately 2.8%, whereas the original YOLOv7 model stabilizes at approximately 3.9%. The improved model demonstrated clear superiority, as its loss value was significantly lower than that of the unimproved model, affirming its strong performance.

**Figure 6:**
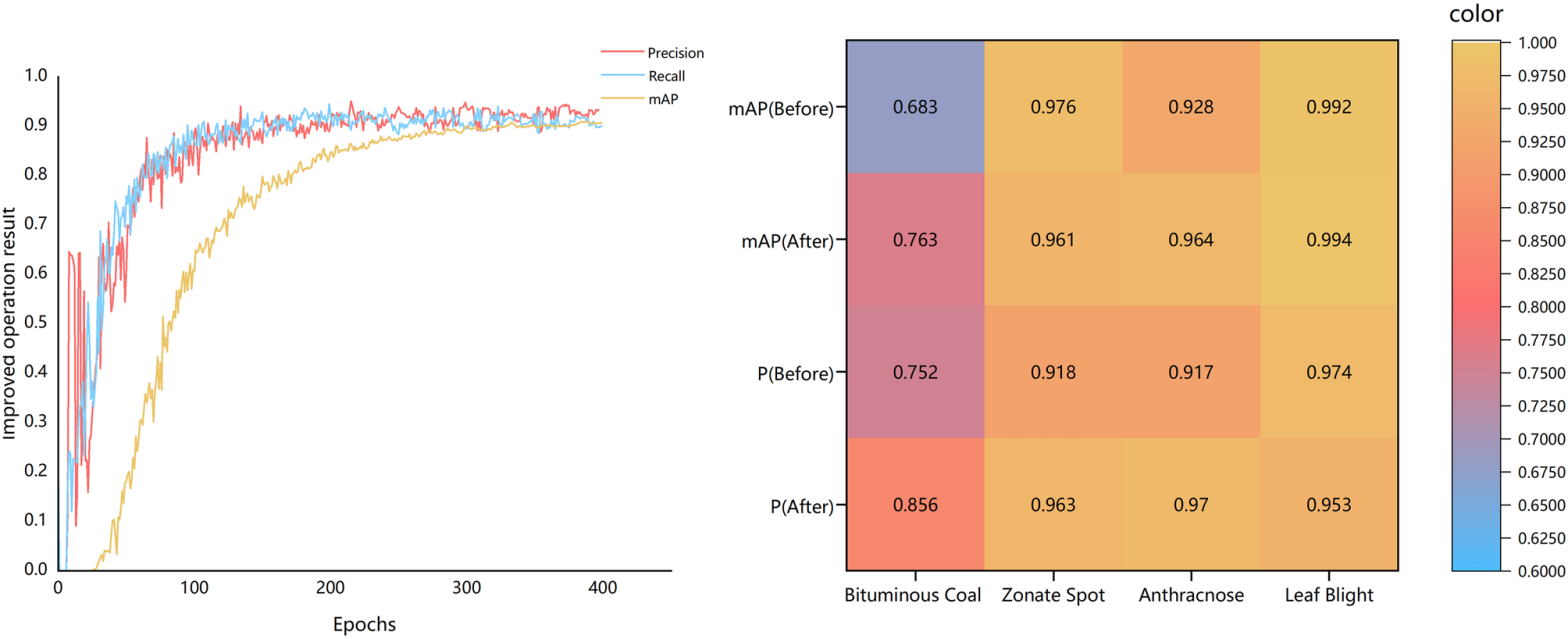
Results of improved model accuracy P, R and mAP (left), Comparison of P and mAP before and after improvement (right).

To comprehensively assess the model’s strengths and weaknesses, three key metrics, namely the P value, R value, and mAP, were selected to encompass a holistic evaluation of the model’s classification performance across different target categories. AP reflects the average accuracy of a specific category across varying IoU thresholds. AP provides a comprehensive evaluation of the model’s positional and predictive accuracy. The AP value is contingent on the model’s accuracy and recall rate and is essentially the area under the Precision-Recall Curve for a particular category. In this curve, the horizontal axis signifies the recall rate, while the vertical axis represents the accuracy rate. mAP represents the average value across all categories’ AP scores, offering a consolidated assessment of the model’s overall performance. It serves as a pivotal criterion for assessing deep learning models, offering a comprehensive measure of detection model performance. These metrics collectively play an instrumental role in gauging the model’s relative merits and shortcomings. The running result of the model is shown in Fig. 6.

The graphical representation above unmistakably illustrates that the improved YOLOv7 model exhibits superior learning capabilities and enhanced recognition accuracy. Evidently, the P value, R value, and mAP indices demonstrate a sustained and pronounced upward trajectory. As the number of training rounds increased, these metrics gradually stabilized, entering a state of steady fluctuations. According to the model training results, the improved YOLOv7 model showed a good recognition effect and accurately identified four different types of tea diseases.

In order to further explore the advantages of the improved model in this paper, the following ablation experiments were carried out. The specific results were shown in Table 2. The results showed that after replacing the backbone network of the YOLOv7 model, the total number of parameters of the model was reduced by 15.2 M, which achieved the purpose of lightweight improvement of the model, and the precision and recall rate were slightly improved. After adding the dual-layer routing attention mechanism, the model’s ability to extract disease features was obviously enhanced, and the model volume was slightly increased. Replacing the SIoU loss function, the overall performance of the model was improved. It could be seen that the improvements proposed in this paper had a positive effect on the identification of disease types in the complex background of tea.

**Table 2:** Comparison of ablation experimental results.

Algorithm	MobileNeXt	Biformer	SIoU	P	R	mAP	Total param/M	Model volume /MB
1				0.89	0.88	0.895	37.21	74.8
2	√			0.91	0.879	0.906	22.21	59.6
3	√	√		0.922	0.903	0.917	24.33	60.3
4	√	√	√	0.935	0.899	0.921	24.33	50.11

**Figure 7:**
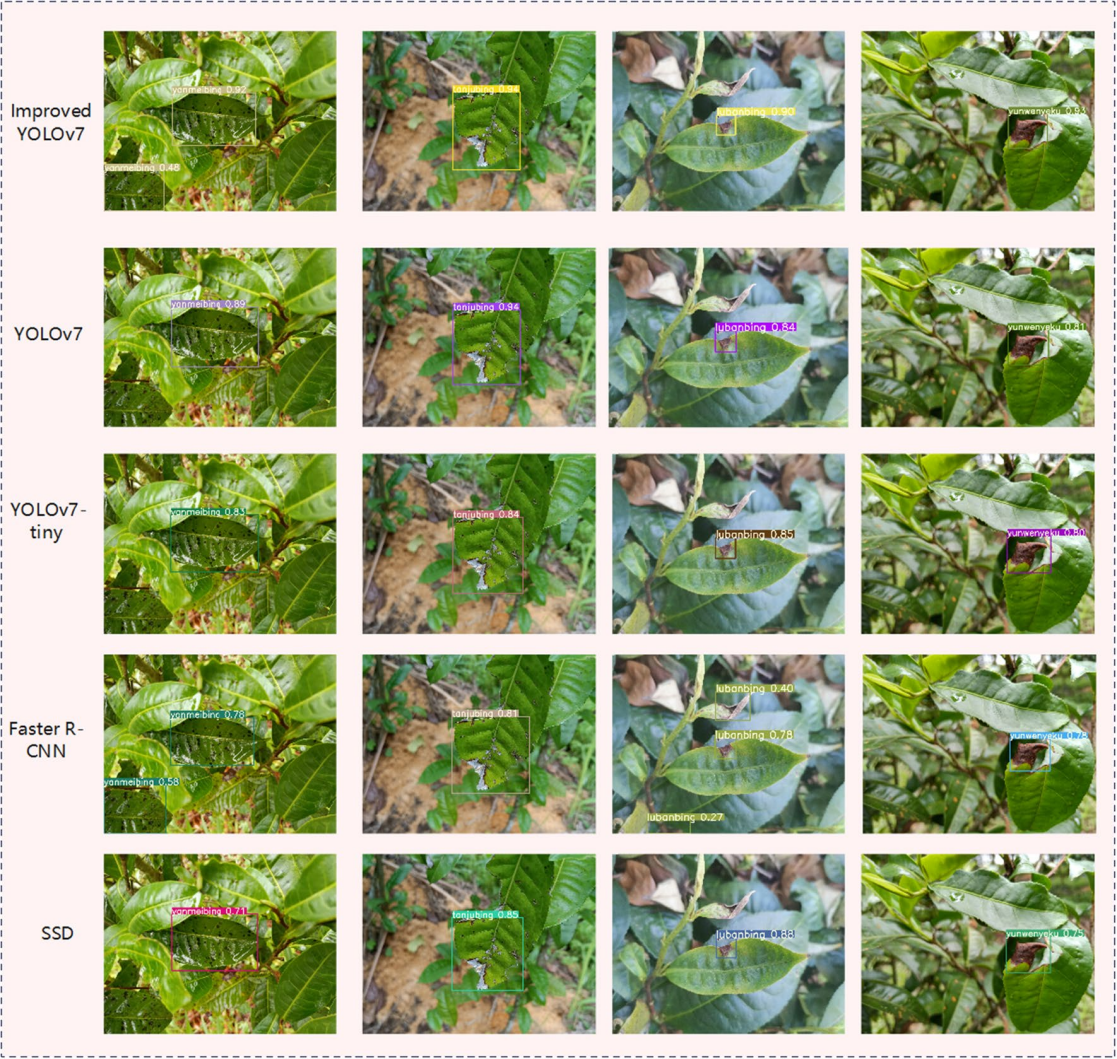
Results of disease identification.

To further validate the competitive edge of the enhanced model proposed in this study, the detection and recognition performance of various models was compared for tea bituminous coal disease, anthracnose, leaf spot, and leaf blight. The comparative findings are presented in following Table 3. At the same time, to ensure result reliability, the original YOLOv7, YOLOv7-tiny, Faster R-CNN, and SSD network models were trained and tested on an identical dataset, while maintaining uniformity in the training platform configuration. The recognition accuracy of the four diseases before and after improvement is shown in Fig. 7.

**Table 3:** Comparison of results of different models.

Model	Precision	Recall	mAP	Examination Speed/FPS	Total param/M	Model volume /MB
ImprovedYOLOv7	0.935	0.899	0.921	66.37	24.33	60.5
YOLOv7	0.89	0.88	0.895	41.96	37.21	74.8
YOLOv7-tiny	0.852	0.839	0.873	45.02	22.02	53.2
Faster R-CNN	0.803	0.811	0.816	7.34	137.09	99.6
SSD	0.824	0.835	0.864	17.42	26.28	83.7

Based on the outcomes detailed in the above table, it was evident that the improved YOLOv7 model attained mAP surpassed that of YOLOv7, YOLOv7-tiny, Faster R-CNN, and SSD by 2.9, 5.5, and 6.6 percentage points, respectively. Compared with other comparison models, the improved YOLOv7 model achieved good recognition results in both P value and R value. While model volume and total param of the improved YOLOv7 model may be marginally inferior to those of the YOLOv7-tiny model, the improved YOLOv7 model boasts mAP increased of 5.5%. The total parameters of the improved YOLOv7 model were reduced by 34.61% compared with the original model, which was the smallest among the five models. Consequently, improved YOLOv7 model detection performance was superior and more practical for real-world applications in tea garden disease detection.

## Discussion

In this study, an improved algorithm based on the YOLOv7 model was proposed to solve the problems of low disease recognition rate and slow model response speed in the tea garden. The algorithm used MobileNeXt to lightweightly improve the backbone network of the YOLOv7 model, successfully reducing the parameter number and calculation cost of the YOLOv7 network model, and providing the basis for further deployment and practical application of the model. At the same time, a dual-layer routing attention visual converter was introduced to retain the characteristics of fine-grained regions, increase the model’s ability to extract disease features, and effectively improve the accuracy of the model. The SIoU function was used to replace the original loss function to achieve the purpose of accelerating the convergence of the model. After improvement, the model showed good recognition performance compared with the original model. The recognition accuracy of four tea diseases was improved. Additionally, the final total loss of the improved YOLOv7 model was always below 2.8%, which was 1.1% lower than that of the original YOLOv7 network. The volume of the model was reduced by 19.12%, the number of total parameters of the model was reduced 34.61%, and the detection speed was increased by 58.17%.

At the same time, there were still some shortcomings in this study. There were still other types of diseases in the complex environment of the tea garden. In the follow-up study, the quality and type of collected data will be further enriched, and the accuracy and generalization ability of the model will be improve. This will provide support for the deployment of the model on the mobile terminal and truly contributed to the tea garden disease detection and intelligent tea industry.

## Data Availability

Our relevant data are allowed by Yunnan Agricultural University and related bases. All data generated or analysed during this study are available in the Github repository. Links to the code and datasets are provided in the below hyperlinked text. Code of Improved YOLOv7 project: https://github.com/anqi99/yolov7.git.

## References

[1] Bhagat M, Kumar D (2023). Stability analysis of mathematical model for spread of pest in tea plant by Rkm-4 and Abm-2. J. Differ. Equ. Appl..

[2] Lee LK, Foo KY (2013). Recent advances on the beneficial use and health implications of Pu-Erh Tea. Food Res. Int..

[3] Sanlier N (2018). A minireview of effects of white tea consumption on diseases. Trends Food Sci. Technol..

[4] Bhagat M, Kumar D (2023). Performance enhancement of kernelized svm with deep learning features for tea leaf disease prediction. Multimed. Tools Appl..

[5] Bhagat M, Kumar D (2023). Efficient feature selection using bows and surf method for leaf disease identification. Multimed. Tools Appl..

[6] Sankaranarayanan AC, Veeraraghavan A, Chellappa R (2008). Object detection, tracking and recognition for multiple smart cameras. Proc. IEEE.

[7] Bhagat M, Kumar D (2022). A comprehensive survey on leaf disease identification & classification. Multimed. Tools Appl..

[8] Wang H, Gu J, Wang M (2023). A review on the application of computer vision and machine learning in the tea industry. Front. Sustain. Food Syst..

[9] Xing W (2022). Suitability evaluation of tea cultivation using machine learning technique at town and village scales. Agronomy.

[10] Cui Q, Yang B, Liu B, Li Y, Ning J (2022). Tea category identification using wavelet signal reconstruction of hyperspectral imagery and machine learning. Agriculture..

[11] Maria SK, Maria SK (2022). Cauliflower disease recognition using machine learning and transfer learning. Smart Systems: Innovations in Computing: Proceedings of SSIC 2021.

[12] Bhagat M, Kumar D, Kumar S (2023). Bell pepper leaf disease classification with Lbp and Vgg-16 based fused features and Rf classifier. Int. J. Inf. Technol..

[13] Anami BS, Malvade NN, Palaiah S (2020). Deep learning approach for recognition and classification of yield affecting paddy crop stresses using field images. Artif. Intell. Agric..

[14] Tiwari D, Tiwari D (2020). Potato leaf diseases detection using deep learning. 2020 4th International Conference on Intelligent Computing and Control Systems (ICICCS).

[15] Bhagat M, Kumar D, Kumar S (2023). Optimized transfer learning approach for leaf disease classification in smart agriculture. Multimed. Tools Appl..

[16] Soeb MJA (2023). Tea leaf disease detection and identification based on Yolov7 (Yolo-T). Sci. Rep..

[17] Zhang S (2023). Edge device detection of tea leaves with one bud and two leaves based on shufflenetv2-Yolov5-lite-E. Agronomy.

[18] Chen J, Wang W, Zhang D, Zeb A, Nanehkaran YA (2021). Attention embedded lightweight network for maize disease recognition. Plant Pathol..

[19] Lee SH, Lin SR, Chen SF (2020). Identification of tea foliar diseases and pest damage under practical field conditions using a convolutional neural network. Plant Pathol..

[20] Xiang, Z. & Chunman, Y. E-Mobilenext: Face Expression Recognition Model Based On Improved Mobilenext. *Optoelectron. Lett.* **20**, 122–128 (2024).

[21] Jia L (2023). Mobilenet-Ca-Yolo: An improved Yolov7 based on the mobilenetv3 and attention mechanism for rice pests and diseases detection. Agriculture.

[22] He, J. *et al.* Pest Recognition in Microstates State: An Improvement of Yolov7 Based On Spatial and Channel Reconstruction Convolution for Feature Redundancy and Vision Transformer with Bi-Level Routing Attention. *Front. Plant Sci. ***15**, 1327237 (2024). 10.3389/fpls.2024.1327237 PMC10877420 38379942

[23] Ye, R., Gao, Q., Qian, Y., Sun, J. & Li, T. Improved Yolov8 and Sahi Model for the Collaborative Detection of Small Targets at the Micro Scale: A Case Study of Pest Detection in Tea. *Agronomy.* **14**, 1034 (2024).

[24] Zhu J (2023). Fire detection in ship engine rooms based on deep learning. Sensors.

[25] Hu G, Yang X, Zhang Y, Wan M (2019). Identification of tea leaf diseases by using an improved deep convolutional neural network. Sustain. Comput. Inform. Syst..

[26] Wang Y, Wang H, Xin Z (2022). Efficient detection model of steel strip surface defects based on Yolo-V7. IEEE Access.

[27] Wei G (2023). Bfd-Yolo: A Yolov7-based detection method for building façade defects. Electronics.

[28] Dong C, Jiang X (2023). An intelligent detection method for optical remote sensing images based on improved Yolov7. Comput. Mater. Contin..

[29] Beazley K (2008). Operation sandglass: Old history, contemporary lessons. Secur. Chall..

[30] Kasagi A, Tabaru T, Tamura H, Kasagi A, Tabaru T, Tamura H (2017). Fast algorithm using summed area tables with unified layer performing convolution and average pooling. 2017 IEEE 27th International Workshop on Machine Learning for Signal Processing (MLSP).

[31] Wang, M. *et al.* Yolo-T: Multitarget Intelligent Recognition Method for X-Ray Images Based On the Yolo and Transformer Models.* Applied Sciences*. **12**, 11848 (2022).

[32] Chen J, Mai H, Luo L, Chen X, Wu K, Chen J, Mai H, Luo L, Chen X, Wu K (2021). Effective feature fusion network in bifpn for small object detection. 2021 IEEE International Conference On Image Processing (ICIP).

[33] Zheng, Z. et al. 2020 Distance-Iou Loss: Faster and Better Learning for Bounding Box Regression. Proceedings of the AAAI conference on artificial intelligence, 12993–13000, (2020).

[34] Gevorgyan, Z. Siou Loss: More Powerful Learning for Bounding Box Regression. Arxiv Preprint. Arxiv:2205.12740 (2022).

[35] Chen, J. *et al.* Apple Inflorescence Recognition of Phenology Stage in Complex Background Based On Improved Yolov7. Comput. Electron.* Agric*. **211**, 108048 (2023).

